# A Case of Male Breast Cancer Patient with CHEK2*1100delC Mutation

**DOI:** 10.7759/cureus.8972

**Published:** 2020-07-02

**Authors:** Quan D Nguyen, Anahita Tavana, Florentino Saenz Rios, Flavia E Posleman Monetto, Angelica S Robinson

**Affiliations:** 1 Radiology, University of Texas Medical Branch, Galveston, USA; 2 Diagnostic Radiology, University of Texas Medical Branch, Galveston, USA; 3 Radiology, University of Texas Medical Branch, Galveston , USA

**Keywords:** male breast cancer, mbc, mammogram, breast ultrasound, ct scan, gynecomastia, invasive ductal carcinoma, idc, chek2, 1100delc variant

## Abstract

Male breast cancer (MBC) is a rare disease that accounts for less than one percent of all breast cancers. The association between *BRCA1* and *BRCA2* mutations and MBC has been well-established; recent data suggest that CHEK2 1100delC heterozygosity is also associated with an increased risk of MBC. Herein, we present the case of a 47-year-old male who was initially diagnosed with bilateral symmetric gynecomastia on a diagnostic mammogram performed for right breast palpable lump. Sixteen months after his diagnosis of gynecomastia, he presented with enlarging right breast palpable lumps and underwent a diagnostic mammogram and breast ultrasound. Ultrasound-guided biopsies were performed on the right breast mass and axillary lymphadenopathy. Pathology revealed right breast invasive ductal carcinoma (IDC) and right axillary metastatic lymphadenopathy. Subsequent genetic testing found CHEK2*1100delC mutation. This case report focuses on the presentation, diagnosis, and management of breast cancer, as well as long-term cancer screening in the setting of *CHEK2* mutation in a relatively young male patient.

## Introduction

Male breast cancer (MBC) is a rare occurrence, comprising less than 1% of cancer in men and less than 0.2% of all cancer-related mortality among men. Risk factors for the development of breast cancer in men include alcohol use, as well as factors that could lead to a hyperestrogenic state, such as obesity, cirrhosis, testicular injury, and undescended testes [1]. Moreover, several breast cancer susceptibility genes are known risk factors for breast cancer, including *BRCA 1* and *BRCA2* mutations. In addition, *CHEK2* mutations have been shown to predispose both women and men to specific types of breast cancer. CHEK2 protein is a cell-cycle checkpoint kinase, responsible for phosphorylating the tumor suppressor proteins p53 and BRCA1, which subsequently leads to either cell cycle arrest or activation of DNA repair proteins. Loss-of-function variants in *CHEK2* are known to be pathogenic as they result in impaired DNA repair and genomic instability. More specifically, the 1100delC variant results in the deletion of one nucleotide from exon 11 of the CHEK2 mRNA, causing a frameshift at codon 367; this forms a premature stop codon, creating a disrupted or absent protein product and abolishing the kinase function of CHEK2 [2]. *CHEK2* mutation is inherited in an autosomal dominant manner [3].

The association between *CHEK2* mutation and female breast cancer has been extensively studied in the past. According to one meta-analysis by Weischer et al., CHEK2 1100delC variant can increase the risk of breast cancer three- to five-fold among women; in addition, women with a family history of breast cancer are estimated to have a 37% cumulative risk of breast cancer at age 70 years, while those with no family history of breast cancer are estimated to have a 21% cumulative risk [4]. Another meta-analysis by Yang *et al*. confirmed the significant association between the CHEK2 1100delC variant and female breast cancer risk, particularly in familial breast cancer cases among Caucasians [5].

*CHEK2 *mutation has also been associated with an increased risk of MBC, although the association has not been as well-studied as in female breast cancer [6]. From the articles that have studied this association between *CHEK2 *mutation and MBC thus far, however, one can conclude that the prevalence of CHEK2 mutation is higher in some countries compared to the others. Higher rates of *CHEK2 *mutation have been reported in studies from Northern European countries, mainly the Netherlands; the mutation, however, seems to be rare in Australia, Spain, and Ashkenazi Jews [7]. Therefore, the evaluation of the association between CHEK2 mutation and MBC has been limited so far due to both the rarity of the MBC cases as well as the significant differences in the CHEK2*1100delC population frequencies [6]. Herein, we present a case of MBC with *CHEK2 *mutation.

## Case presentation

**Figure 1:**
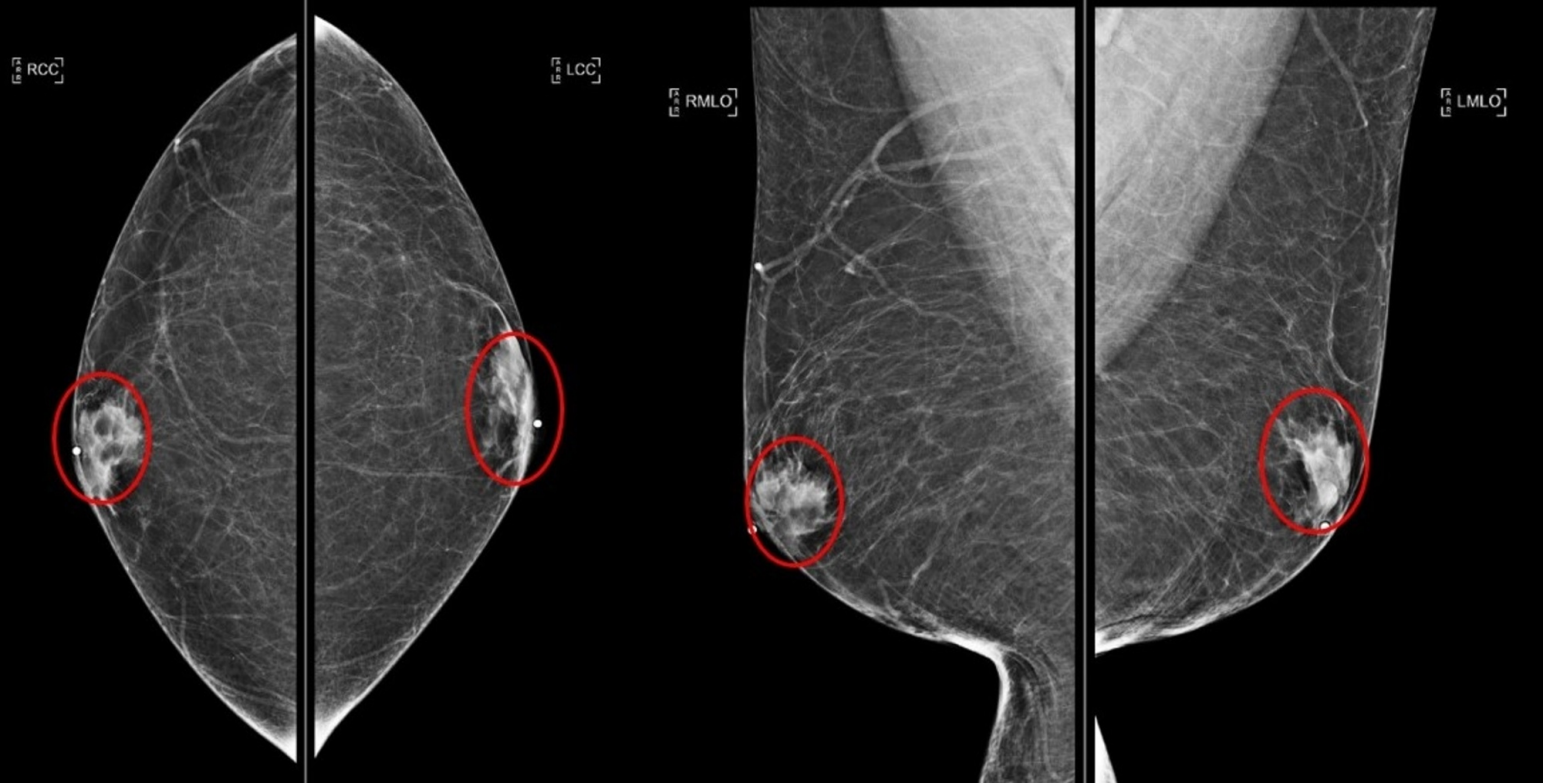
Initial diagnostic mammogram for right breast lump Bilateral breast symmetric gynecomastia (red circles); craniocaudal (left image) and mediolateral oblique (right image) views.

**Figure 2:**
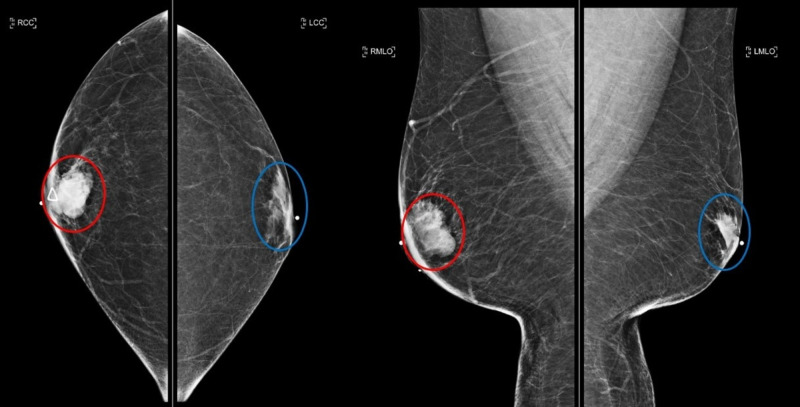
Subsequent diagnostic mammogram sixteen months later for enlarging right breast lump Right breast irregular mass measuring 35 mm (red circle); left breast gynecomastia (blue circle); craniocaudal (left image) and mediolateral oblique (right image) views.

**Figure 3:**
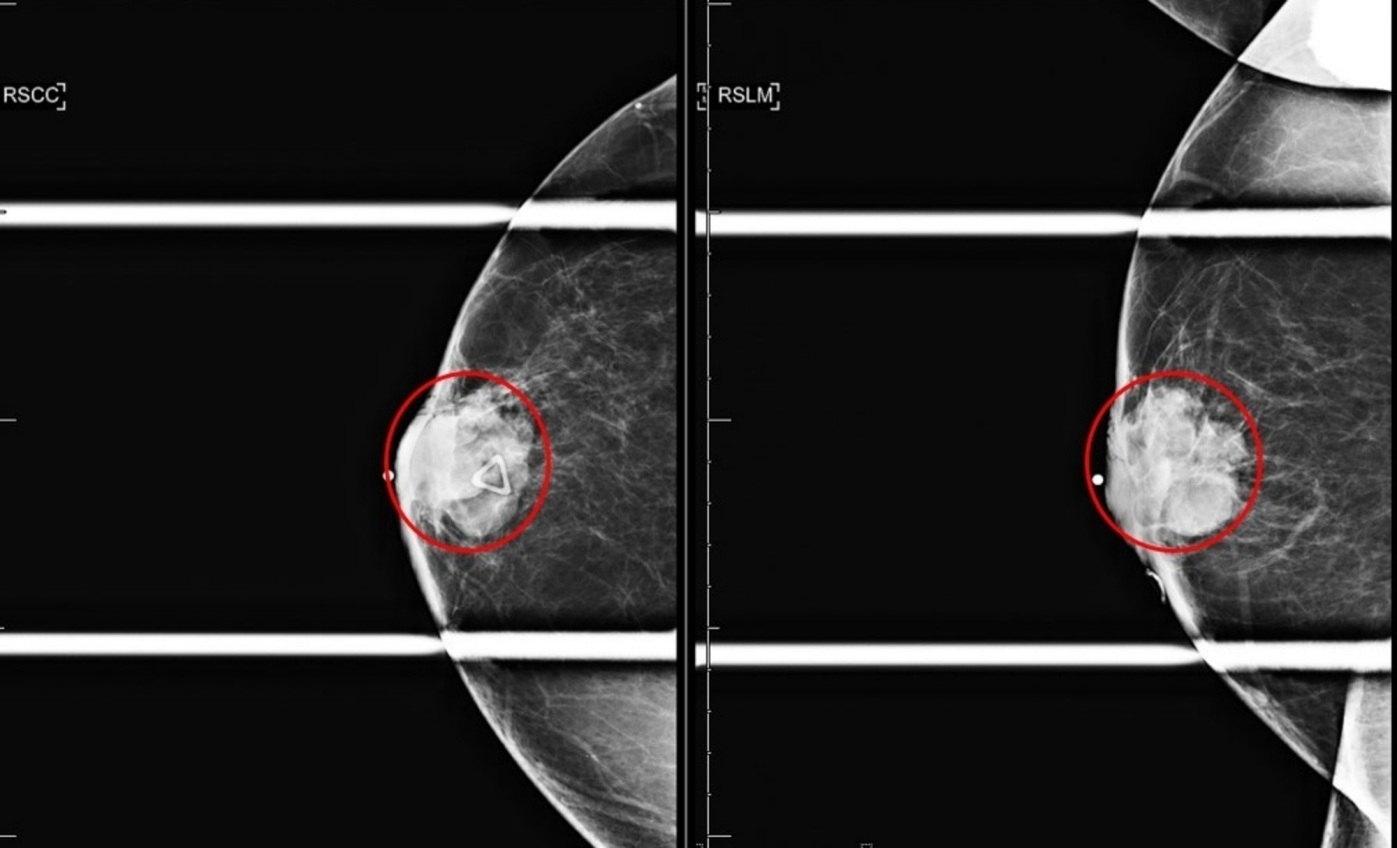
Right breast diagnostic mammogram spot compression CC and LM Views Right breast irregular mass measuring 35 mm (red circle); craniocaudal (left image) and lateromedial (right image) views.

**Figure 4:**
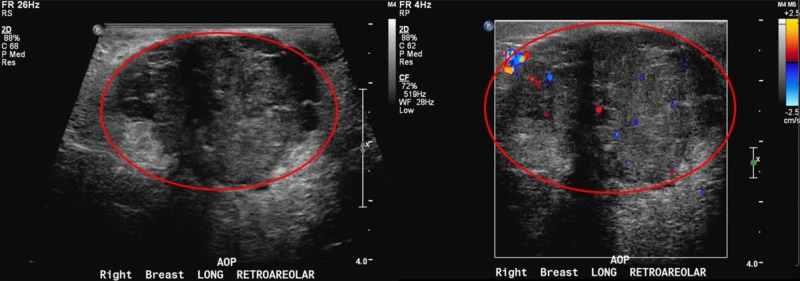
Right breast diagnostic ultrasound Right breast irregular mass with internal vascular color flow measuring 35 x 26 x 24 mm (red circle).

**Figure 5:**
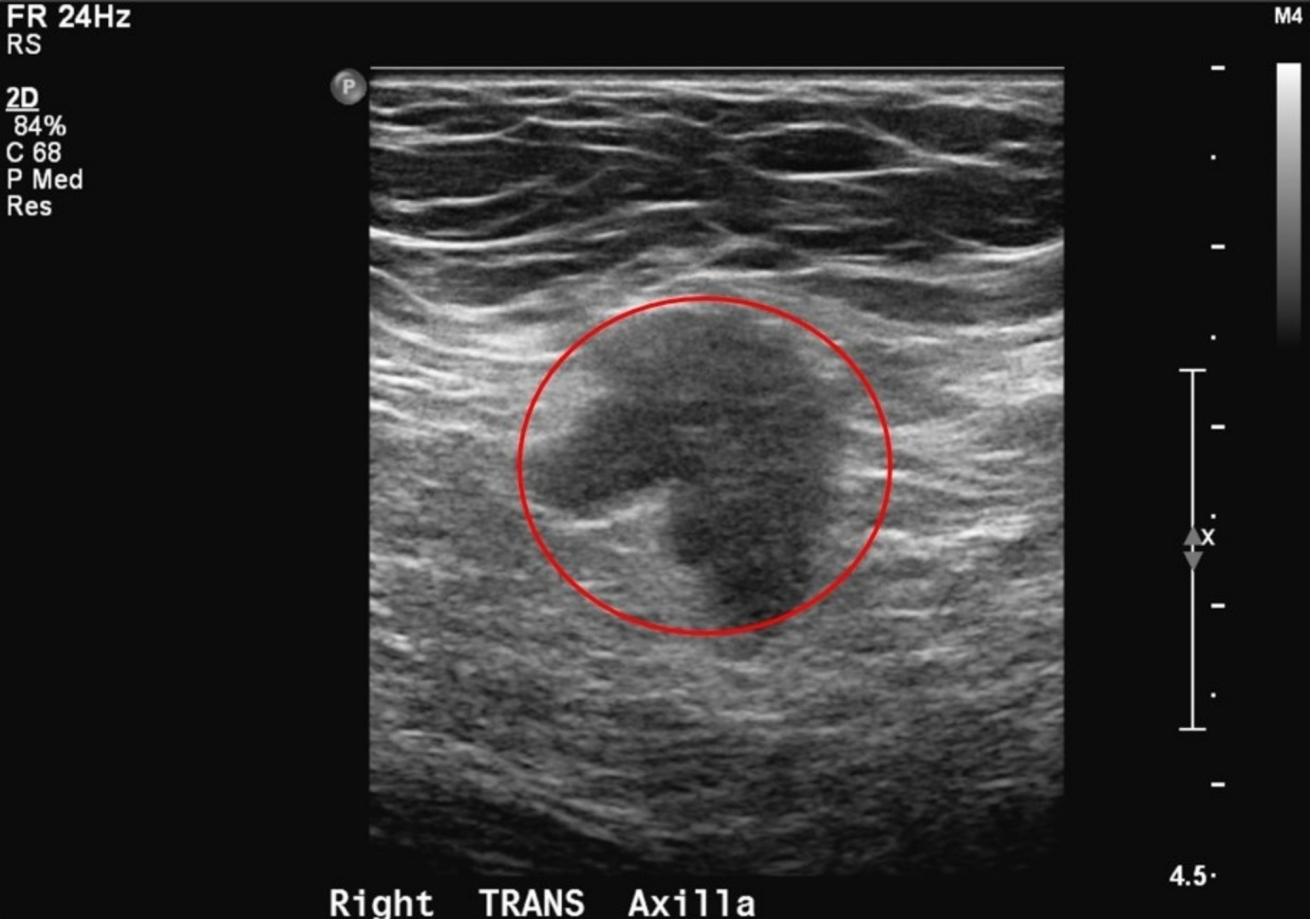
Right axilla diagnostic ultrasound Morphologically abnormal axillary lymph node measuring 19 mm (red circle).

**Figure 6:**
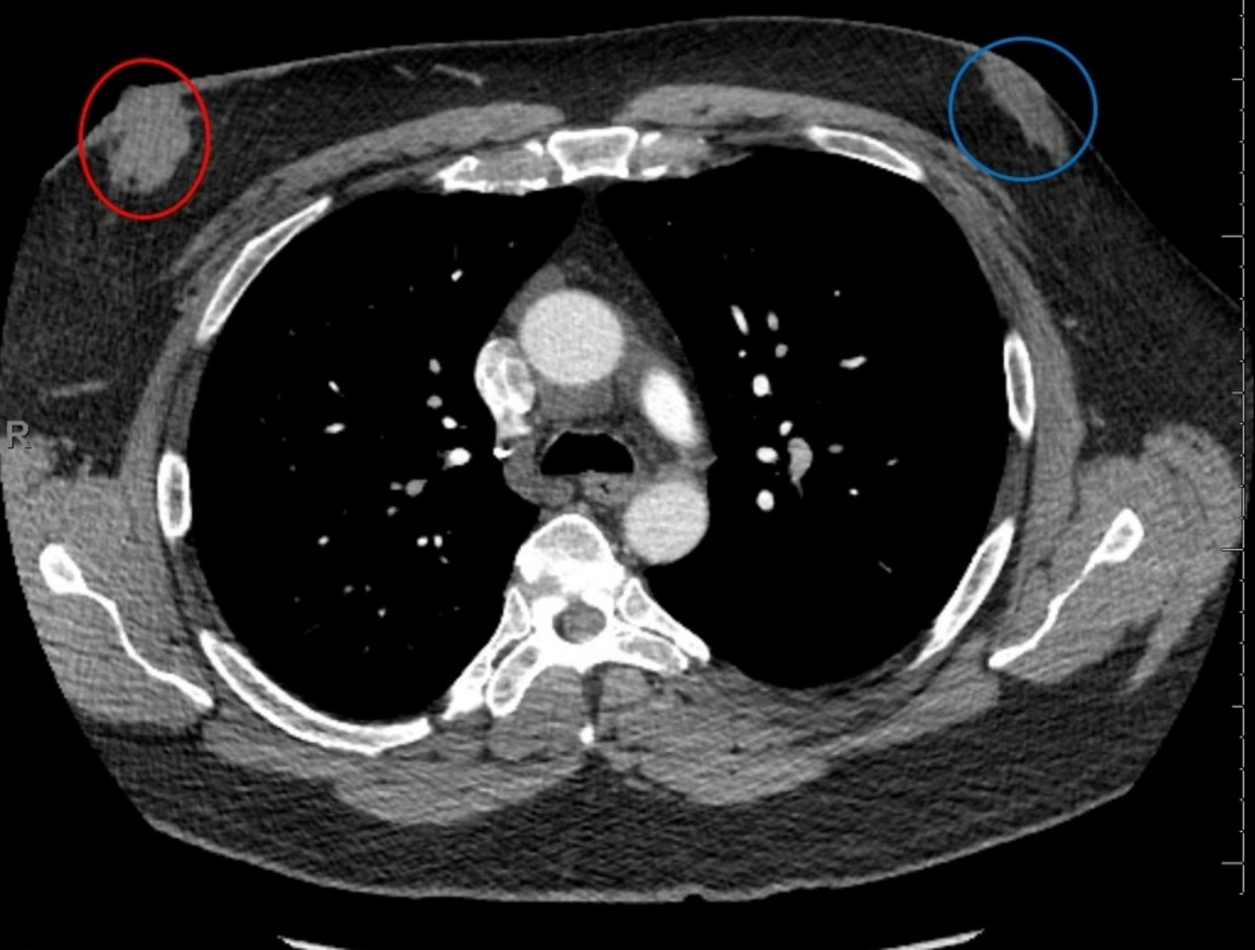
CT chest Right breast biopsy-proven malignancy (red circle); left breast gynecomastia (blue circle); no evidence of intra-thoracic metastases. CT, computed tomography

The patient is a 47-year-old male with a past medical history of bilateral gynecomastia that was first detected via a mammogram in November 2018 with no evidence of malignancy present on mammogram at the time (Figure 1). In addition, he has a past surgical history of Roux-en-Y gastric bypass in 2007 with revision done in 2013, with his most recent body mass index being 37 kg/m^2^. His social history is positive for a 45-pack-year history of cigarette smoking, which he has now quit, as well as heavy alcohol abuse until one year ago, which he has recently cut down to one shot of whiskey a day. Sixteen months after his diagnosis of gynecomastia, the patient presented with a two-month history of right retroareolar palpable mass along with new-onset tingling, numbness, and pain of the right breast. On exam, the patient had a right-sided subareolar mass measuring 5 x 3 cm that was mobile from chest wall but fixed to skin, along with color changes of the nipple. In addition, he had a right axillary mass that was mobile and approximately 3 cm in size. A diagnostic mammogram and breast ultrasound showed an irregular right retroareolar mass along with an abnormal-appearing right axillary lymph node, both of which were highly suggestive of malignancy (BI-RADS 5) (Figures 2, 3). The breast mass measured 35 x 26 x 24 mm and the axillary lymph node measured 19 x 17 x 18 mm on ultrasound (Figures 4, 5). Ultrasound-guided core biopsy of the right-sided retroareolar was performed with pathology revealing cT2, cN1, cM0, Grade 3, ER/PR positive, HER2 negative, Clinical Stage IIB invasive ductal carcinoma of the right breast. Additional metastatic workup imaging with computed tomography (CT) scan of chest, abdomen, and pelvis was negative (Figure 6).

The patient underwent a modified radical mastectomy with sentinel lymph node mapping; post-mastectomy pathology report confirmed pT2, pN1a, M0, Grade 3, ER/PR positive, Ki-67 50%, HER2 FISH not amplified, Path Stage IIA invasive ductal carcinoma, with negative surgical margins. The patient is currently followed by medical oncology with the plan for Adriamycin/Cyclophosphamide-Taxol (AC-T) regimen with curative intent, followed by evaluation for post-mastectomy radiation therapy by radiation oncology and at least five years of endocrine therapy with Tamoxifen.

In addition, given that he is a male with breast cancer, he was recommended to undergo genetic counseling, as he met the National Comprehensive Cancer Network (NCCN) criteria for hereditary cancer testing [8]. The patient elected to proceed with multi-gene panel testing, which revealed a CHEK2 mutation, specifically the 1100delC heterozygote variant. Regarding his pedigree, patient-reported paternal English and Irish ancestry and maternal German ancestry. His family history was also remarkable for colon cancer in his maternal grandmother in her early 50s. The patient has one biological son with no significant past medical history.

## Discussion

Our case presented a 47-year-old male who was diagnosed with right-sided invasive ductal carcinoma in the setting of *CHEK2 *mutation, sixteen months after he was found to have bilateral symmetric gynecomastia on a mammogram. The peak age for MBC is 71 years old, while our patient is only 47 years old. Although our patient has gynecomastia, which is found in 6% to 38% of males affected by breast cancer, gynecomastia per se is not a risk factor of MBC [9]. The risk factors for breast cancer development in our patient included obesity and heavy alcohol use, in addition to *CHEK2 *mutation; the combination of all these risk factors could have led to a relatively early onset of breast cancer in this patient.

Male patients presenting with breast symptoms such as breast enlargement, nipple discharge, breast pain, and palpable lump are usually concerned about the underlying cause and whether or not the symptoms are a manifestation of breast cancer. The most common cause of breast symptoms in male patients is gynecomastia, especially in younger patients, as MBC has a later onset on average in comparison to gynecomastia, which can present at any age [10]. Detection of MBC often occurs at a later stage compared to female breast cancer due to the rarity of breast cancer in men as well as a lower index of suspicion at initial presentation; therefore, more advanced features such as larger tumor size, the involvement of lymph nodes, and metastases at the time of diagnosis are more common in male versus female breast cancer [1]. According to NCCN guidelines, for men presenting with bilateral breast enlargement consistent with gynecomastia or pseudo-gynecomastia secondary to the buildup of excess adipose tissue, clinical management would suffice. However, for men presenting with palpable breast mass or thickening, bloody nipple discharge, or presumed asymmetric gynecomastia on clinical exam, initial evaluation involves diagnostic mammogram and possible breast ultrasound. Imaging would be followed by either core-needle biopsy or clinical management depending on the presence or absence of suspicious findings, respectively [11].

Although not as extensively studied as in female breast cancer, recent studies have explored the link between *CHEK2 *mutation and MBC. According to a study by Wasielewski *et al*., CHEK2 1100delC is associated with an increased risk for MBC particularly in the Dutch population, with the average age at diagnosis of 69 years; most of the CHEK2 1100delC MBC cases in the study were also ER/PR positive compared to CHEK2 1100delC negative cases [6]. Another study by Cybulski *et al*. investigated the link between *CHEK2 *mutation and ER status in women with early-onset breast cancer and found a four-fold increased risk of ER-positive breast cancer, suggesting that Tamoxifen could be used as chemoprevention in the setting of confirmed *CHEK2 *mutation [12].

According to a study by Marjanka *et al*., breast tumor characteristics of *CHEK2 *mutation carriers do not differ from those of non-carriers, with no significant differences observed in terms of tumor morphology, angioinvasion, or lymph node involvement [13]. Therefore, the same course of treatment is generally followed for MBC in *CHEK2 *carriers as non-carriers. Due to the rarity of MBC, recommendations regarding its management is based upon the results of clinical trials focusing on female breast cancer. Surgery is usually the first step in the treatment of MBC. Modified radical mastectomy is generally preferred over radical or total mastectomy due to a lower rate of complications despite no significant difference in cancer recurrence or survival. Breast-conserving surgery (lumpectomy) is another option but is usually not feasible due to the central location of most male breast tumors, as well as the small breast volume in men. Another important part of the surgical treatment of invasive MBC is axillary lymph node dissection [14]. In addition, the indications for post-surgical radiotherapy in MBC are the same as for female breast cancer [15]. Adjuvant radiation therapy is associated with improved survival, particularly in patients with positive lymph nodes [16]. In terms of systemic therapy, men with advanced breast cancer should be managed similarly to women. Since the majority of male breast cancers are ER-positive, at least 5-10 years of Tamoxifen therapy is recommended. In MBC, however, if Tamoxifen is contraindicated, a gonadotropin-releasing hormone (GnRH) analog is used alongside aromatase inhibitors to promote adequate estradiol suppression [15]. Adjuvant chemotherapy is also associated with improved survival and is recommended in male patients with positive axillary lymph node status [14]. The same chemotherapeutic agents that are recommended in invasive female breast cancer should be used for invasive MBC [1]. Our patient, with ER/PR positive intraductal carcinoma and positive axillary lymph node, underwent a modified radical mastectomy and is scheduled to undergo a course of AC-T chemotherapy and receive post-surgical radiotherapy and at least five years of Tamoxifen treatment.

In addition to breast cancer, *CHEK2 *mutation can predispose to other types of cancer. According to a study by Näslund-Koch *et al*., *CHEK2* heterozygosity is associated with a 15-82% increased risk for any cancer; they propose that this mutation could be a susceptibility allele for any cancer, especially given its function as a protein kinase involved in DNA repair and stability [17]. Another study by Kleibalova *et al*. suggests that second primary cancers, such as cancers of colon, thyroid, lung, and kidney, are more frequent in *CHEK2 *mutation carriers compared to non-carriers [18]. In addition, two meta-analyses, one by Liu *et al*. and another by Xiang *et al*., have explored the link between the CHEK2 mutation and colorectal cancer and have found an increased risk for colorectal cancer among *CHEK2 *mutation carriers [3,19]. For this reason, NCCN guidelines suggest colonoscopy screening every five years beginning at age 40 if there are no first-degree relatives diagnosed with colorectal cancer or every five years beginning at age 40 or 10 years prior to the age of a first-degree relative with colorectal cancer [20]. Our patient was also recommended to schedule a colonoscopy in the future following his oncology treatment.

Moreover, since *CHEK2 *mutation is inherited in an autosomal dominant fashion, our patient was informed that other family members may also carry this pathogenic variant. He was informed that children of *CHEK2 *carriers also have a 50% risk to carry the pathogenic variant with a 50% chance for his son to carry the familial *CHEK2 *pathogenic variant. Since it is unclear whether the *CHEK2 *pathogenic variant was maternally or paternally inherited at this point, testing his maternal and paternal relatives can help determine which family members are also at risk of carrying the pathogenic variant. The patient was encouraged to discuss the results with his family members and to talk to them regarding seeking genetic counseling to clarify their risk.

## Conclusions

In most cases, when a male presents with a breast lump, the diagnosis will be gynecomastia. However, MBC is also on the differential, and thus, assessment with a diagnostic mammogram and breast ultrasound can be helpful. MBC is uncommon, and it typically occurs in elderly men with a peak age of 71 years old. In this case, the patient is only 47 years old. Although he is relatively younger than the peak age for MBC, his genetic predisposition with the *CHEK2 *mutation explains his diagnosis of MBC at a relatively younger age. The patient also has gynecomastia, but gynecomastia is not a risk factor for breast cancer according to the current literature. Breast cancer genetic testing is important after diagnosis of MBC to provide further information to the patient as well as his family members. Due to the association of *CHEK2 *mutation with other malignancies, further cancer surveillance is recommended for patients with this mutation.
